# Percutaneous Coronary Intervention With Rotational Atherectomy in the Elderly Presenting With an Acute Coronary Syndrome: The First Successful Case in Mauritania

**DOI:** 10.7759/cureus.111018

**Published:** 2026-06-17

**Authors:** Soueid Ahmed Sidi Mhamed, Sidi Mohamed Limame, Mohamed Issa Elkharchi, Abdesselam Daty, El Haj Ahmed Ne

**Affiliations:** 1 Department of Cardiology, National Cardiology Center, Nouakchott, MRT

**Keywords:** acute coronary syndrome, calcified coronary lesions, elderly, percutaneous coronary intervention, rotational atherectomy

## Abstract

Percutaneous coronary intervention with rotational atherectomy is a revascularization procedure for calcified coronary lesions. Coronary calcifications reflect an advanced stage of atherosclerosis. Rotational atherectomy is identified as an advanced plaque modification strategy in densely calcific and non-dilatable coronary lesions. This concept was described in the late 1980s and approved for use in calcific coronary lesions in 1993. It has demonstrated its effectiveness and safety in routine use. However, rotational atherectomy in elderly patients in an acute setting is poorly studied. This case report describes the first successful rotational atherectomy in Mauritania performed on an elderly patient with acute coronary syndrome and encourages the use of this technique to prepare calcified lesions in our sub-Saharan African context.

## Introduction

Percutaneous coronary intervention with rotational atherectomy is a revascularization procedure for calcified coronary lesions. Coronary calcifications are observed in 25% of patients eligible for percutaneous coronary intervention [1]. They reflect an advanced stage of atherosclerosis with several hypotheses for initiation, including the formation of calcium phosphate crystals following the death of inflammatory cells in the atherosclerosis, releasing apoptotic bodies and necrotic debris that serve as nucleation sites, circulating nucleation complexes, reduced local expression of mineralization inhibitors, and differentiation of pericytes or vascular smooth muscle cells inducing bone formation [2]. Coronary calcifications are common in diabetics, the elderly, and those with renal insufficiency, and during percutaneous coronary intervention, they pose risks of stent thrombosis, stent underexpansion, and procedural complications [3].

Rotational atherectomy is identified as an advanced plaque modification strategy in densely calcific and non-dilatable coronary lesions. This concept was described in the late 1980s and approved for use in calcific coronary lesions in 1993. It has demonstrated its effectiveness and safety in routine use [4]. Rotational atherectomy selectively resects non-compliant atherosclerotic tissue using a differential cutting mechanism while preserving elastic vascular tissue to allow balloon dilation and stent deployment [5,6].

Rotational atherectomy during acute coronary syndrome is relatively contraindicated, but recent studies demonstrate its feasibility with good short-term results [7]. Data on rotational atherectomy in elderly patients with acute coronary syndrome are limited, particularly in Africa, where conventional percutaneous coronary intervention is still restricted to certain referral centers [8,9].

The National Cardiology Center in Mauritania (sub-Saharan Africa) is a high-volume tertiary center with an interventional cardiology team that performs over 1,000 percutaneous coronary interventions annually. In this case report, we describe the first successful percutaneous coronary intervention with rotational atherectomy in an elderly patient with acute coronary syndrome in Mauritania.

## Case presentation

A 92-year-old patient with no prior medical history presented with typical angina that had been developing for 24 hours prior to admission. He was hemodynamically stable with a blood pressure of 140/80 mmHg, a heart rate of 100 beats per minute, and a respiratory rate of 21 breaths per minute. The physical examination revealed no abnormalities. The electrocardiogram showed sinus rhythm with ST-segment depression in the apicolateral leads. Troponin kinetics were rising. Transthoracic echocardiography was normal with a left ventricular ejection fraction of 65%. The diagnosis of acute coronary syndrome without ST-segment elevation was made. Coronary angiography (Video 1) revealed a heavily calcified lesion of 90% of the proximal-middle left anterior descending artery and a 40% stenosis of the ostium of the left circumflex artery. The right coronary artery was dominant without any significant lesions.

**Video 1:** Coronary angiography Coronary angiography reveals a heavily calcified lesion of the proximal-middle anterior interventricular artery.

Percutaneous coronary intervention was performed via the radial artery. The left main coronary artery was selectively cannulated with a 6F EBU 3.5 guiding catheter. The Rotablator® device (Boston Scientific, Maple Grove, Minnesota, United States) was used to prepare the calcified lesion of the left anterior descending artery. The rota wire was inserted at the level of the left anterior descending artery calcified lesion, and rotational atherectomy was performed with a 1.25 mm burr, then at 1.5 mm, at 175,000 rpm (Video 2). The lesion was then crossed by the All Star 0.014"×190 cm guidewire, followed by predilation with successive balloons: 2.5×15 mm at 18 atm, 3×10 mm at 20 atm, and 3×20 mm at 20 atm.

**Video 2:** Rotational atherectomy Rotational atherectomy was used for the preparation of the calcified lesion.

Stenting was performed with a 3.5×38 mm drug-eluting stent (Resolute Integrity®, Medtronic, Dublin, Ireland) at 12 atm (Video 3).

**Video 3:** Stenting Stenting was performed with a 3.5×38 mm drug-eluting stent (Resolute Integrity®).

Postdilation was performed with a 3.5×10 mm balloon at 28 atm. The successful stenting of the left anterior descending artery using rotational atherectomy yielded satisfactory results with a TIMI 3 flow without procedural complications (Video 4).

**Video 4:** Final result Final result with a TIMI 3 flow.

The patient was discharged from the hospital 24 hours after the percutaneous coronary intervention, and the 30-day follow-up was favorable with no cardiovascular events.

## Discussion

In this case report, we describe the first percutaneous coronary intervention with rotational atherectomy performed in Mauritania. This case illustrates a rotational atherectomy performed on an elderly patient admitted for acute coronary syndrome with a heavily calcified lesion of the left anterior descending artery. The success of this procedure is attributable to the expertise of the local interventional cardiology team.

Percutaneous coronary intervention of coronary calcifications presents a major challenge due to the risk of stent underexpansion and device fracture, even with the latest generation of stents, and a poor long-term prognosis. Preparing these calcified lesions allows for good stent apposition and reduces the risk of thrombosis and stent restenosis [10].

Rotational atherectomy is a preparation technique that aims for the physical removal of calcified atheroma using a diamond-encrusted elliptical metallic burr (1.25-2.5 mm) rotated at high speeds. The differential cutting method employed during rotational atherectomy allows for the preferential removal of inelastic calcified plaques, which are unable to stretch under the burr's action, while preserving healthy elastic tissue. The microdebris produced by rotational atherectomy is small enough (approximately 5 µm) to cross the coronary microcirculation and be eliminated by the reticuloendothelial system. Optimal rotational atherectomy minimizes the risk of procedural complications such as dissection, perforation, slow flow, and no reflow [5,11].

The feasibility and safety of rotational atherectomy are being studied by several registries. The European multicentre Euro4C registry demonstrated a high success rate of percutaneous coronary intervention with rotational atherectomy performed via the radial approach, with a low rate of procedural complications. Furthermore, the one-year major adverse cardiovascular event rate was relatively high [12]. Another long-term follow-up study suggested that the high mortality rate after rotational atherectomy is not attributable to the procedure itself, but rather to the initially high risk profile of the patients [13]. The Rota China registry demonstrated that performing rotational atherectomy in patients with severely calcified lesions is associated with a high success rate and good short- and long-term outcomes [14]. Rotational atherectomy in very elderly patients can be performed safely, with hospital outcomes similar to those in younger patients [8]. Rotational atherectomy in high-risk patients with acute coronary syndrome is technically feasible and safe. However, long-term follow-up of these patients has revealed a higher incidence of adverse cardiovascular events compared to patients with stable coronary artery disease [15].

In our patient, despite his high cardiovascular risk and the performance of the rotational atherectomy in an emergency context, the success of this procedure and the good short-term result reflected the safety of this procedure and its effectiveness in treating severely calcified lesions.

## Conclusions

Percutaneous coronary intervention in elderly patients with acute coronary syndrome carries procedural risks related to severely calcified lesions and a high cardiovascular risk. Routine preparation of severely calcified lesions by rotational atherectomy has yielded good short- and long-term results. However, rotational atherectomy in elderly patients in an acute setting is poorly studied. This case report describes the first successful rotational atherectomy in Mauritania performed on an elderly patient with acute coronary syndrome and encourages the use of this technique to prepare calcified lesions in our sub-Saharan African context. Furthermore, this case reinforces the idea of ​​​​the feasibility and safety of rotational atherectomy in very elderly patients in the context of acute coronary syndrome.
